# The Inoculability of the Tuberculosis of Mammiferæ on the Gallinaceæ

**Published:** 1897-01

**Authors:** 


THE INOCULABILITY OF THE TUBERCULOSIS OF MAMMIFERÆ ON THE GALLINACEÆ. Cadiot, Gilbert, and Roger have shown that the higher temperature of birds is not the principal factor in the resistance to the tuberculosis of mammiferæ. They have tried to bring about a condition of low temperature by plucking the feathers, practising partial starvation, keeping the feet in water, etc., but without satisfactory result. Daily injections of a drachm of antipyrin, three times a day, continued for three weeks, reduced the temperature from 38° to 40°—that is, to the temperature of the rodents; but the twelve fowls subject to this *régime* remained free from tuberculosis. After having cited three new cases of transmission of the tuberculosis of mammals to fowls the authors sum up the positive facts which they have obtained in this way since the beginning of their investigations: Of a total of 86 fowls, 7 have contracted human tuberculosis. The bacillus of the mammiferæ sometimes retains its original characteristics during its passage through the bird—that is to say, it is still very virulent for the cobaye (cobæa?), and cannot be applied anew to the fowl; sometimes, however, it is sensibly modified in the manner of an aviary bacillus, showing itself fitted to live anew in the bird; in one case the bacillus modified in this way preserved the faculty of taking a new lease of life in the dog—a faculty little characteristic of aviary virus. Later researches of the authors have confirmed them in their former opinion that the human and aviary bacilli are not two species, but two races of the same species which have become acclimated upon different lands, so to speak.—*Annales de Méd. Vét.*, March, 1896.

